# The Mechanism and Clinical Outcome of patients with Corona Virus Disease 2019 Whose Nucleic Acid Test has changed from negative to positive, and the therapeutic efficacy of Favipiravir: A structured summary of a study protocol for a randomised controlled trial

**DOI:** 10.1186/s13063-020-04430-y

**Published:** 2020-06-05

**Authors:** Jiawen Li, Chi Zhang, Zhao Wu, Guiqiang Wang, Hong Zhao

**Affiliations:** Department of Infectious Disease, Center for Liver Disease, Peking University First Hospital, No.8 Xishiku Street, Xicheng District, Beijing, China

**Keywords:** COVID-19, Randomised controlled trial, protocol, Favipiravir

## Abstract

**Objectives:**

A variety of possible mechanisms can make the nucleic acid test of patients who meet the discharge conditions positive again, including reinfection, reactivation of the original virus, lack of strict discharge criteria, new infection, and so on. Different reasons will correspond to different prevention and control measures. We will enroll patients who are discharged after treatment, whose nucleic acid test has changed from negative to positive during the screening visit, regardless of the severity of the symptoms, to investigate the mechanism, clinical outcome and therapeutic efficacy with Favipiravir patients with Corona virus Disease 2019. Favipiravir is an anti-viral agent that selectively and potently inhibits the RNA-dependent RNA polymerase, it has been used for treatment of some life-threatening infections such as Ebola virus, Lassa virus and rabies. Its therapeutic efficacy has been proven in these diseases.

**Trial design:**

This is a multi-center, two arm, open label, parallel group, randomized controlled trial.

**Participants:**

Eligibility criteria:

Inclusion criteria:

1. Adults 18 to 80 years, male or female.
2. After the first diagnosis and treatment of COVID-19, the nucleic acid test of respiratory specimens such as sputum or nasopharyngeal swabs, has been negative for two consecutive times (sampling time interval of at least 24 hours), in accordance with the COVID-19’s diagnosis and treatment Plan (7th Edition), discharged.
3. During screening visit (follow-up after discharge), The nucleic acid test of COVID-19 is positive in any one of the following samples: sputum, throat swabs, blood, feces or other specimens. Regardless of whether or not they had symptoms and the severity of symptoms.
4. Volunteer to participate in the research and sign the Informed Consent Form.

Exclusion Criteria:

1. Allergic to Favipiravjr;
2. Pregnant or lactating women
3. Uncontrolled diseases of the blood and cardiovascular system, liver or kidney.
4. History of mental disorders, drug abuse or dependence;
5. Researchers consider it inappropriate for adults to participate;
6. Participating in other clinical studies.

Loss to Follow up:

Cases that do not complete the clinical trial program will be regarded as lost to follow up. Including the withdrawal of patients by themselves (such as poor compliance, etc.), or the withdrawal of patients ordered by the researcher (those who need other drugs which affect the judgment of the curative effect, and those who need to stop taking drugs for severe adverse events)

Study setting:

The participating hospitals are some of the designated hospitals that have been or may be admitting patients who meet the eligibility criteria, mainly in Hubei, Shenzhen, Anhui and Beijing. Participants will be recruited from these 15 hospitals: Wuhan Pulmonary Hospital, Hubei; Jinyintan Hospital of Wuhan, Hubei; Ezhou Central Hospital, Hubei; The Second People's Hospital of Fuyang, Anhui; The First Affiliated Hospital of USTC, Anhui; Beijing Youan Hospital, Beijing; Capital Medical University Beijing Institute of Hepatology, Beijing; Ezhou Hospital of Traditional Chinese Medicine, Hubei; Zhongnan Hospital of Wuhan University, Hubei; The Fifth Hospital of ShiJiazhuang, Hebei; Jinan Infectious Diseases Hospital, Shandong; Public Health Clinical Center of Chengdu, Sichuan; Wuxi No.5 People’s Hospital, Jiangsu; The Third People’s Hospital of Shenzhen, Guangdong; The First Affiliated Hospital of Bengfu Medical College, AnHui.

**Intervention and comparator:**

Favipiravir group (experimental): Favipiravir 1600mg each dose, twice a day on the 1st day; 600mg each dose, twice a day from the 2nd to the 7th day, Oral administration, the maximum number of days taken will be no more than 14 days plus routine treatment for COVID-19.

Regular treatment group (control): Treatments other than Antiviral drugs can be given. Routine treatment for patients with the corona virus will be administered, this includes oxygen therapy, drugs that reduced phlegm and relieve cough, including thymosin, proprietary Chinese medicine, etc.

**Main outcomes:**

Primary Outcome Measures: Viral nucleic acid test negative [Time Frame: 5 months]: Subjects who tested negative for nucleic acid from sputum or nasopharyngeal swabs for two consecutive times (sampling time interval of at least 24 hours).

Secondary Outcome Measures: Clinical cure [Time Frame: 5 months]:

1. Body temperature returned to normal for more than 3 days;
2. Lung image improved.
3. Clinical manifestation improved;
4. The viral nucleic acid test of respiratory specimens was negative for two consecutive times (sampling time interval of at least 24 hours).

**Randomization:**

The central randomization system (Interactive Web Response Management System), will be used to randomly divide the subjects into the experimental group and the control group according to the ratio of 2:1. In this study, block randomization will be used, in blocks of 6.

**Blinding (masking):**

This is an open label trial. Trial participants, investigators, care givers, outcome assessors, and date analysts are not blinded to group assignment.

**Numbers to be randomised:**

210 patients are expected to be enrolled and allocated according to the ratio of 2 (Favipiravir group, n=140): 1(regular treatment group, n=70).

**Trial Status:**

Protocol version number 3.0, 10^th^ April 2020

First Patient, first visit 17^th^ March 2020; recruitment end date anticipated June 1, 2020.

**Trial registration:**

ClinicalTrials.gov, NCT04333589, April 3, 2020. Registered April 3, 2020.

**Full protocol:**

The full protocol is attached as an additional file, accessible from the Trials website (Additional file 1). In the interest in expediting dissemination of this material, the familiar formatting has been eliminated; this Letter serves as a summary of the key elements of the full protocol.

## Supplementary information

**Additional file 1.** Full Protocol.

## Data Availability

The datasets generated during and/or analyzed during the current study will be available from the author (e-mail: john131212@126.com) on reasonable request.

