# Supplementary material for: The Mechanism and Clinical Outcome of patients with Corona Virus Disease 2019 Whose Nucleic Acid Test has changed from negative to positive, and the therapeutic efficacy of Favipiravir: A structured summary of a study protocol for a randomised controlled trial
Source: Trials. 2020 Jun 5;21:488. doi: 10.1186/s13063-020-04430-y (PMC7273382; doi:10.1186/s13063-020-04430-y)
Supplement: Supplementary file 1 — Additional file 1. Full Protocol. [file 13063_2020_4430_MOESM1_ESM.docx]

**COVID-19患者核酸“复阳”**

**发生机制、临床转归和治疗干预的研究**

| **版 本 号：** | V 3.0 |
| --- | --- |
| **版本日期** | 2020年4月10日 |
| **项目申办单位：** | 北京大学第一医院 |
| **项目负责人：** | 王贵强 |
| **联系电话** | 13911405123 |
|  |  |
|  |  |
|  |  |
|  |  |

**保密声明**

本方案中所包含的所有信息的所有权归申办单位，仅提供给研究者、合作研究者、伦理委员会和监督管理部门等相关机构和人员审阅。在未得到申办单位书面批准情况下，除了在与可能参加本试验的受试者签署知情同意书时，向其做必要的解释外，严禁将任何信息告知与本试验无关的第三方。

**申办单位名称和地址：**

名 称： 北京大学第一医院（牵头负责单位）

地 址： 北京市西城区西什库大街8号

联 系 人： 王贵强

邮 编： 100034

手 机： 13911405123

E –mail： john131212@sina.com

**研究机构名称和地址：**

名 称：武汉肺科医院

联 系 人：彭鹏

电 话：18971570937

名 称： 武汉金银潭医院

联 系 人： 张定宇

手 机： 13507117929

名 称： 鄂州市中心医院

联 系 人： 喻军华

手 机： 13908688619

名 称：深圳市第三人民医院

联 系 人：张政

手 机：13927461302

联 系 人：李国军

手 机：13691944938

名 称：阜阳市第二人民医院

联 系 人：韩明锋

手 机：13955881280

武汉大学中南医院（雷神山）

联 系 人： 王行环

手 机：

石家庄市第五医院

联 系 人： 郑欢伟

手 机：

名 称：济南市传染病医院

联 系 人：张纵

手 机：

名 称：成都市公共卫生临床中心

联 系 人：曾义岚

手 机：

名 称：无锡市第五人民医院

联 系 人：邱源旺

手 机：

名 称：蚌埠医学院第一附属医院

联 系 人：李伟

手 机：

名 称：首都医科大学附属北京佑安医院

联 系 人：金荣华

手 机：

名 称：中国科学技术大学附属第一医院

联 系 人：

手 机：

名 称：鄂州市中医医院

联 系 人：陈新胜

手 机：

**研究人员**

研究负责人： 王贵强

研究设计者：王贵强，赵鸿

研究参加者： 王贵强，赵鸿，阙呈立，王鹤，彭鹏，张定宇，喻军华，朝明锋，王行环，郑欢伟，张纵，曾义岚，邱源旺，张政，李国军，李伟，金荣华，陈新胜。

**研究日期**

计划研究起始日期：2020年4月1日

计划研究完成日期：2020年9月15日

**签名**

专题负责人： 王贵强 日期： 年 月 日

中心负责人： 日期： 年 月 日

委托方负责人： 日期： 年 月 日

**合规申明**

本研究遵照中国国家食品药品监督管理局颁布的《药物临床试验质量管理规范（GCP 现行版）》进行，并遵守世界医学大会赫尔辛基宣言（第 18 届世界医学协会联合大会，赫尔辛基，芬兰，1964 年 6 月）以及修订版。研究方案遵守地方法规， 与知情同意书的文档一起提交给伦理委员会审批批准后获得伦理批件。

伦理委员会的批准文件以书面的形式送交研究者，然后再由研究者将批准文件（原件或副本）提供给研究申办方。

筛选开始前，研究者对每个受试者解释了本次临床试验的目的、方法、益处和潜在的风险，并获得了临床试验的受试者签署的知情同意书。由临床试验的受试者本人（或法律监护人）签名和注明签署日期的知情同意书由研究者和受试者各自妥善保存一份。

在临床研究过程中，任何与临床研究安全性相关的问题，如临床研究方案的变更（本试验过程中未变更方案）以及临床研究中的严重不良事件，均应及时向伦理委员会报告。

临床研究结束时提交报告给伦理委员会。

**目录**

[方案摘要 7](#_Toc37434991)

[一、研究背景 9](#_Toc37434992)

[二、研究目的和治疗终点 9](#_Toc37434993)

[2.1研究目的 9](#_Toc37434994)

[2.2主要终点 9](#_Toc37434995)

[2.3次要终点 9](#_Toc37434996)

[三、研究方法 9](#_Toc37434997)

[3.1研究设计 9](#_Toc37434998)

[3.2研究的持续时间 10](#_Toc37434999)

[3.3研究对象 10](#_Toc37435000)

[3.4无效病例的剔除标准 10](#_Toc37435001)

[3.5退出标准 11](#_Toc37435002)

[3.6研究计划的终止标准 11](#_Toc37435003)

[3.7脱落标准 11](#_Toc37435004)

[3.8随机化 11](#_Toc37435005)

[3.9研究干预 11](#_Toc37435006)

[3.9.1研究药物 11](#_Toc37435007)

[3.9.2治疗方案 12](#_Toc37435008)

[3.9.3合并用药 12](#_Toc37435009)

[3.10疗效评估 12](#_Toc37435010)

[3.11安全性评估 12](#_Toc37435011)

[3.12访视计划 12](#_Toc37435012)

[3.12.1筛选访视 18](#_Toc37435013)

[3.12.2治疗期访视 18](#_Toc37435014)

[3.12.3随访期访视 19](#_Toc37435015)

[四、数据管理和质量保证 19](#_Toc37435016)

[五、统计分析 20](#_Toc37435017)

[5.1样本量的确定 20](#_Toc37435018)

[5.2统计分析集 20](#_Toc37435019)

[5.3统计分析方法 20](#_Toc37435020)

[六、受试者保护：知情同意、收益风险、保密、利益冲突 21](#_Toc37435021)

[6.1伦理方面的考虑 21](#_Toc37435022)

[6.2试验方案的审批 22](#_Toc37435023)

[6.3知情同意过程和知情同意书文本 22](#_Toc37435024)

[6.4受试者获益和风险 22](#_Toc37435025)

[6.5保密声明 22](#_Toc37435026)

[七、不良事件、严重不良事件的评估及处理 22](#_Toc37435027)

[7.1不良事件观察 22](#_Toc37435028)

[7.2 AE分级 22](#_Toc37435029)

[7.3 AE与研究药物之间关系的判断 23](#_Toc37435030)

[7.4 AE的记录 23](#_Toc37435031)

[7.5 严重不良事件的报告和处理 23](#_Toc37435032)

[八、研究质量控制 25](#_Toc37435033)

[8.1 研究培训 25](#_Toc37435034)

[8.2 数据核查 25](#_Toc37435035)

[8.3 质量控制报告 25](#_Toc37435036)

[九、研究进度安排 25](#_Toc37435037)

# 方案摘要

| **研究名称** | COVID-19患者核酸“复阳”发生机制、临床转归和治疗干预的研究 |
| --- | --- |
| **研究设计** | 前瞻性、多中心、开放、随机对照研究 |
| **试验目的** | 初探COVID-19患者核酸“复阳”的发生机制、观察患者临床转归及法匹拉韦干预的效果 |
| **研究分组及**  **治疗方案** | 受试者按照2:1进行随机化分组，分别给予法匹拉韦+基础治疗和基础治疗：   - 法匹拉韦治疗组：第1天给予法匹拉韦片，1次1600mg，BID；第2天到第7天，1次600mg，BID。治疗7天后，研究者可根据受试者具体情况决定是否继续服用法匹拉韦，治疗时间最长不超过14天。 - 基础治疗组：可给予除克立芝、磷酸氯喹、硫酸羟氯喹、阿比朵尔、可利霉素之外的治疗。 |
| **计划中心数量** | 8家 |
| **计划招募受试者数量** | 210例 |
| **研究周期** | 5个月 |
| **入选标准** | （1）年龄18～80周岁，男性或女性；  （2）曾确诊为COVID-19，且经治疗后连续两次痰或鼻咽拭子等呼吸道标本核酸检测阴性（采样时间至少间隔24小时）；  （3）筛选访视时痰、咽拭子、血液、粪便等标本COVID-19核酸检测阳性；  （4）自愿参与研究并签署知情同意书。 |
| **排除标准** | （1）对法匹拉韦过敏者；  （2）孕妇或哺乳期妇女；  （3）未稳定控制的血液系统、肝、肾、心脏疾病；  （4）精神异常、药物滥用或依赖史；  （5）研究者认为不适合参与研究；  （6）正在参与其他临床研究。 |
| **主要有效性评价指标** | - COVID-19病毒核酸阴转率 |
| **次要评价指标** | - 临床治愈率（按照国家卫健委发布的诊疗方案试行第七版中定义的出院标准执行）。 |

# 一、研究背景

新型冠状病毒肺炎患者出现复阳的情况是指患者在出院之后核酸的检测再次由阴性变为阳性。目前对于出院的患者出现复阳的原因仍然没有一个定论，这个情况也引起了我国各界的困惑。有专家分析，出现复阳的原因可能是与新型冠状病毒的特性有关。由于新型冠状病毒是一种近期出现的病毒，对于这个病毒的进化我们仍然还未了解，因为对该病毒缺乏深入全面的了解，所以也有可能是造成患者出院后出现复发的现象。有相关的研究发现产生复阳的原因，第二个可能是与细胞免疫功能降低有关。因为患者体内的细胞免疫功能降低，导致病毒在体内清除不够彻底，从而出现复发的情况。还有一个比较重要的原因，可能是在采样以及标本处理检测过程中存在问题。目前对于新型病毒肺炎的确诊主要是通过核酸检测。新冠肺炎患者主要感染的部位是下呼吸道以及肺部。在检测的时候可能会出现假阴性的情况。

由于目前出现复阳的患者越来越多，而对于复阳的原因也越来越难以确定。因此，有必要尽快开展相关研究，以进一步明确复阳患者的传染性、临床转归以及相应的防控和治疗方案等问题。

# 二、研究目的和治疗终点

## 2.1研究目的

初探COVID-19患者核酸“复阳”的发生机制、观察患者临床转归及法匹拉韦干预的效果。

## 2.2主要终点

主要终点指标为经治疗后连续两次痰或鼻咽拭子等呼吸道标本核酸检测阴性（采样时间至少间隔24小时）。

## 2.3次要终点

次要终点指标为临床治愈，即经过治疗后达到以下出院标准：

1.体温恢复正常3天以上；

2.呼吸道症状明显好转；

3.肺部影像学显示急性渗出性病变明显改善；

4.连续两次痰或鼻咽拭子等呼吸道标本核酸检测阴性（采样时间至少间隔24小时）。

# 三、研究方法

## 3.1研究设计

本研究采用前瞻性、多中心、开放、随机、对照研究设计，预计纳入210例受试者，按2:1比例进行随机，分别给予法匹拉韦+基础治疗（n=140）和基础治疗（n=70）：

- 法匹拉韦治疗组：第1天给予法匹拉韦片，1次1600mg，BID；从第2天到第7天，1次600mg，BID。治疗满7天后，研究者可根据受试者具体情况决定是否继续服用法匹拉韦，治疗时间最长不超过14天。
- 基础治疗组：可给予除克立芝、磷酸氯喹、硫酸羟氯喹、阿比朵尔、可利霉素之外的治疗。

## 3.2研究的持续时间

本研究预期持续5个月，包括为期2个月的受试者招募期。

开始研究的日期：2020年4月1日。

最后一名受试者入组的日期：2020年6月1日。

研究结束的日期：2020年9月15日（最后1例合格受试者入选治疗结束后满28天）

数据库锁定时间：2020年9月31日。

## 3.3研究对象

本研究将入选18～80周岁、COVID-19经治疗出院后病毒“复阳”的患者。受试者来自各研究中心收治的患者。各研究中心根据竞争入组原则入选符合本研究所定义的入选、排除标准的受试者。

**纳入标准：**

（1）年龄18～80周岁，男性或女性；

（2）曾确诊为COVID-19，且经治疗后连续两次痰或鼻咽拭子等呼吸道标本核酸检测阴性（采样时间至少间隔24小时）；

（3）筛选访视时痰、咽拭子、血液、粪便等标本COVID-19核酸检测阳性；

（4）自愿参与研究并签署知情同意书。

**排除标准：**

（1）对法匹拉韦过敏者；

（2）孕妇或哺乳期妇女；

（3）未稳定控制的血液系统、肝、肾、心脏疾病；

（4）精神异常、药物滥用或依赖史；

（5）研究者认为不适合参与研究；

（6）正在参与其他临床研究。

## 3.4无效病例的剔除标准

（1）不符合入选标准的病例；

（2）符合排除标准的病例；

（3）未遵照方案要求服用治疗药物的病例；

（4）缺重要检查记录，或重要检查不符合要求的患者，包括：无疗效评估相关检查、无COVID-19病毒核酸检查等。

## 3.5退出标准

（1）受试者发生严重不良事件，根据研究者判断必须中断研究；

（2）受试者怀孕；

（3）试验期间同时参与其他临床研究者；

（4）受试者自己要求退出；

（5）研究者认为需要中止试验。

退出病例应说明原因，并记录于电子病例报告表（eCRF）。有至少1次用药记录和安全性数据的受试者应纳入统计分析。

## 3.6研究计划的终止标准

（1）受试者撤回知情同意书；

（2）受试者出现严重不良事件；

（3）治疗14天后随访满28天。

## 3.7脱落标准

未完成临床试验方案的病例应视为脱落。包括病人自行退出（如依从性差，不愿意继续治疗等）及研究者令其退出（病情严重需合并应用其他药物而影响疗效判断者，重度不良事件需停药者）。脱落病例应说明原因，并将其最后一次主要疗效检测结果转接为最终结果纳入全分析集（FAS）进行统计分析。有一次用药记录均应纳入安全性分析集（SS），进行安全性分析。所有脱落病例应在eCRF中记录脱落原因。

## 3.8随机化

本研究采用中央随机化系统（基于网络的交互式网络应答系统，IWRS）实现随机分组。符合入排标准的受试者按2:1比例随机分为试验组和对照组。本研究采用区组随机化分组方法，区组长度为6。随机化过程由统计和计算机专业人员设定随机分组程序。

## 3.9研究干预

### 3.9.1研究药物

研究药物为法匹拉韦片剂（批件号：2020S00038；批号XXXX）由军事医学研究院毒物药物研究所提供，浙江海正药业有限公司生产，规格为200mg/片，经检验符合质量检验标准。研究中涉及的COVID-19基础治疗药物，可依照各研究中心治疗常规选择品牌，便于提高治疗的依从性。

### 3.9.2治疗方案

签署知情同意书且符合入选排除标准的受试者将随机分为对照组（基础治疗组）和试验组（法匹拉韦+基础治疗组）。

基础治疗：根据受试者的治疗需要，可使用除克立芝、磷酸氯喹、硫酸羟氯喹、阿比朵尔、可利霉素之外的治疗药物。

试验组-法匹拉韦的用法：口服给药，第1天，1次1600mg，1日2次；从第2天到第7天，1次600mg，1日2次。之后，研究者可根据受试者具体情况决定是否继续给予法匹拉韦，治疗时间不超过14天。

治疗结束后，受试者将进入为期28天的随访阶段。

### 3.9.3合并用药

随机化后的治疗期间，受试者不可合并使用克立芝、磷酸氯喹、硫酸羟氯喹、阿比朵尔、可利霉素。

## 3.10疗效评估

**主要疗效指标：**

COVID-19核酸阴转

**次要疗效指标：**

临床治愈（按照国家卫健委发布的诊疗方案试行第七版中定义的出院标准执行）

## 3.11安全性评估

以生命体征、实验室指标（包括外周血细胞分类计数、肝肾功能）、不良事件（AE）、严重不良事件（SAE）作为安全性评估指标。不良事件根据美国卫生及公共服务部2017年公布的常⻅不良事件评价标准（CTCAE）5.0版进行观察和分级。

将用于评价安全性的终点如下：AEs发生率、药物相关的AEs的发生率、死亡发生率、SAEs发生率、药物相关的SAE发生率、导致治疗停止和提前退出研究的AEs发生率。

## 3.12访视计划

根据当前的指南和队列研究所需要的信息，参照访视计划表内容进行受试者的访视，然而研究者也可以根据受试者的病情随时调整访视计划。

本研究访视阶段可分为两阶段。第一阶段为治疗期。入选受试者随机接受或不接受法匹拉韦治疗。完成治疗后，受试者将进入随访阶段，随访至治疗结束后28天，随访期间禁止应用前述合并用药。

**访视计划表**

| **阶 段** | **筛选期** | **治疗期** | | | | | | | | | | | | | | **随访期** | | **提前退出/结束治疗^13^** |
| --- | --- | --- | --- | --- | --- | --- | --- | --- | --- | --- | --- | --- | --- | --- | --- | --- | --- | --- |
| **访 视** | **1** | **2** | **3** | **4** | **5** | **6** | **7** | **8** | **9** | **10** | **11** | **12** | **13** | **14** | **15** | **16** | **17** |  |
| **时 间** | **-2天**～**0天** | **1天^12^** | **2天** | **3天** | **4天** | **5天** | **6天** | **7天** | **8天** | **9天** | **10天** | **11天** | **12天** | **13天** | **14天** | **治疗结束后14天±3天** | **治疗结束后28天±3天** |  |
| 签署知情同意书 | √ |  |  |  |  |  |  |  |  |  |  |  |  |  |  |  |  |  |
| 尿妊娠试验 | √ |  |  |  |  |  |  |  |  |  |  |  |  |  |  |  |  |  |
| 确定入选、  排除标准 | √ | √ |  |  |  |  |  |  |  |  |  |  |  |  |  |  |  |  |
| 填写人口学资料 | √ |  |  |  |  |  |  |  |  |  |  |  |  |  |  |  |  |  |
| 体格检查 | √ | √ |  | √ |  | √ |  | √ |  |  | √ |  |  |  | √ |  |  | √ |
| COVID-19前  既往病史  及其治疗史 | √ |  |  |  |  |  |  |  |  |  |  |  |  |  |  |  |  |  |
| 合并用药与  合并治疗 | √ | √ |  | √ |  | √ |  | √ |  |  | √ |  |  |  | √ | √ | √ | √ |
| 此次因COVID-19入院诊断 | √ |  |  |  |  |  |  |  |  |  |  |  |  |  |  |  |  |  |
| 起病日期 | √ |  |  |  |  |  |  |  |  |  |  |  |  |  |  |  |  |  |
| 前次出院诊断 | √ |  |  |  |  |  |  |  |  |  |  |  |  |  |  |  |  |  |
| 首次核酸阳性  日期 | √ |  |  |  |  |  |  |  |  |  |  |  |  |  |  |  |  |  |
| 前次住院期间应用抗病毒药物时间、名称、疗程 | √ |  |  |  |  |  |  |  |  |  |  |  |  |  |  |  |  |  |
| 前次住院期间应用激素时间、剂量、疗程 | √ |  |  |  |  |  |  |  |  |  |  |  |  |  |  |  |  |  |
| 前次住院期间应用恢复期血浆  时间和量 | √ |  |  |  |  |  |  |  |  |  |  |  |  |  |  |  |  |  |
| 前次住院天数 | √ |  |  |  |  |  |  |  |  |  |  |  |  |  |  |  |  |  |
| 前次出院后隔离方式 | √ |  |  |  |  |  |  |  |  |  |  |  |  |  |  |  |  |  |
| 前次出院后接触其他患者史 |  |  |  |  |  |  |  |  |  |  |  |  |  |  |  |  |  |  |
| 前次出院后服用药物名称、时间、疗程 | √ |  |  |  |  |  |  |  |  |  |  |  |  |  |  |  |  |  |
| 前次出院后加重/新出现的症状及日期 | √ |  |  |  |  |  |  |  |  |  |  |  |  |  |  |  |  |  |
| **有效性观察**^1^ |  |  |  |  |  |  |  |  |  |  |  |  |  |  |  |  |  |  |
| 是否存活 |  | √ |  | √ |  | √ |  | √ |  |  | √ |  |  |  | √ | √ | √ | √ |
| 体温^2^ | √ | √ | √ | √ | √ | √ | √ | √ | √ | √ | √ | √ | √ | √ | √ | √ | √ | √ |
| 生命体征（血压、心率、呼吸频率）^2^ | √ | √ | √ | √ | √ | √ | √ | √ | √ | √ | √ | √ | √ | √ | √ | √ | √ | √ |
| 指端脉氧饱和度检查 | √ | √ |  | √ |  | √ |  | √ |  |  | √ |  |  |  | √ | √ | √ | √ |
| 症状 | √ | √ |  | √ |  | √ |  | √ |  |  | √ |  |  |  | √ | √ | √ | √ |
| 体征 | √ | √ |  | √ |  | √ |  | √ |  |  | √ |  |  |  | √ | √ | √ | √ |
| 血常规检测^3^ | √ | √ |  |  |  |  |  | √ |  |  | √ |  |  |  | √ | √ | √ | √ |
| 尿常规检测 | √ | √ |  |  |  |  |  | √ |  |  | √ |  |  |  | √ | √ | √ | √ |
| 便常规检测 | √ | √ |  |  |  |  |  | √ |  |  | √ |  |  |  | √ | √ | √ | √ |
| 血生化检测^4^ | √ | √ |  |  |  |  |  | √ |  |  | √ |  |  |  | √ | √ | √ | √ |
| CRP检测 | √ | √ |  |  |  |  |  | √ |  |  | √ |  |  |  | √ | √ | √ | √ |
| 降钙素原检测 | √ | √ |  |  |  |  |  | √ |  |  | √ |  |  |  | √ | √ | √ | √ |
| 血乳酸检测 | √ | √ |  |  |  |  |  | √ |  |  | √ |  |  |  | √ | √ | √ | √ |
| 凝血功能检测^5^ | √ | √ |  |  |  |  |  | √ |  |  | √ |  |  |  | √ | √ | √ | √ |
| 心肌酶^6^ | √ | √ |  |  |  |  |  | √ |  |  | √ |  |  |  | √ | √ | √ | √ |
| 血气分析^7^ | √ | √ |  |  |  |  |  | √ |  |  | √ |  |  |  | √ | √ | √ | √ |
| 咽拭子病毒核酸定量 | √ | √ |  | √ |  | √ |  | √ |  |  | √ |  |  |  | √ | √ | √ | √ |
| 便病毒核酸定量 | √ | √ |  | √ |  | √ |  | √ |  |  | √ |  |  |  | √ | √ | √ | √ |
| 痰病毒核酸定量 | √ | √ |  | √ |  | √ |  | √ |  |  | √ |  |  |  | √ | √ | √ | √ |
| 血病毒核酸定量 | √ | √ |  | √ |  | √ |  | √ |  |  | √ |  |  |  | √ | √ | √ | √ |
| 细胞因子检测^8^ | √ | √ |  | √ |  | √ |  | √ |  |  | √ |  |  |  | √ | √ | √ | √ |
| T细胞亚群检测^9^ | √ | √ |  | √ |  | √ |  | √ |  |  | √ |  |  |  | √ | √ | √ | √ |
| 免疫球蛋白检测^10^ | √ | √ |  |  |  |  |  | √ |  |  |  |  |  |  | √ | √ | √ | √ |
| 肺部影像检查^11^ | √ | √ |  |  |  |  |  | √ |  |  |  |  |  |  | √ | √ | √ | √ |
| 出组诊断 |  |  |  |  |  |  |  |  |  |  |  |  |  |  | √ |  |  | √ |
| **安全性观察** |  |  |  |  |  |  |  |  |  |  |  |  |  |  |  |  |  |  |
| 记录不良事件 |  | √ | √ | √ | √ | √ | √ | √ | √ | √ | √ | √ | √ | √ | √ | √ | √ | √ |
| 记录严重不良事件 |  | √ | √ | √ | √ | √ | √ | √ | √ | √ | √ | √ | √ | √ | √ | √ | √ | √ |
| **其他工作** |  |  |  |  |  |  |  |  |  |  |  |  |  |  |  |  |  |  |
| 随机分组 |  | √ |  |  |  |  |  |  |  |  |  |  |  |  |  |  |  |  |
| 药物发放 |  | √ | √ | √ | √ | √ | √ | √ | √ | √ | √ | √ | √ | √ | √ |  |  |  |
| 依从性评价 |  | √ | √ | √ | √ | √ | √ | √ | √ | √ | √ | √ | √ | √ | √ |  |  | √ |

备注：

1. 每天给药前采集生物样本，记录指征；
2. 体温和一般检测在生命体征采集时收集，包括体温、血压（卧位）、心率、呼吸频率；
3. 血常规检查：包括红细胞计数、血红蛋白、红细胞压积、白细胞计数、单核细胞计数及百分比、淋巴细胞计数及百分比、中性粒细胞计数及百分比、嗜酸性粒细胞计数及百分比和嗜碱性粒细胞计数及百分比；
4. 血生化检查：包括丙氨酸转氨酶、天冬氨酸转氨酶、乳酸脱氢酶、肌酸激酶、肌酸激酶同工酶、ｒ-谷氨酰转肽酶、总胆红素、直接胆红素、总蛋白、白蛋白、碱性磷酸酶、尿素、肌酐、钾、钠、氯、葡萄糖、尿酸、总胆固醇、甘油三酯；
5. 凝血功能检测包括：凝血酶时间、凝血酶原时间、活化部分凝血酶时间、纤维蛋白原、国际标准化比值；
6. 心肌酶在血生化中收集；
7. 血气分析根据病情需要随时检测，如果指氧饱和度在95%以上不检测血气分析；
8. 细胞因子检测（统一检测）：包括TNF-α、IL-2、IL-4、IL-6、IFN-α、IFN-β、IFN-γ；
9. 淋巴细胞亚群检测（统一检测）：包括CD3+占比和绝对值、CD4+占比和绝对值、CD8+占比和绝对值；
10. 免疫球蛋白检测：包括IgE、IgM、IgG、IgA、IgD；
11. 胸部影像学检查：胸部CT；
12. 第1天检查项目在随机前进行，可以用随机化前2日内的实验室及影像学检查结果；
13. 提前退出和提前结束治疗者需检查；提前结束治疗为：体温正常3天，间隔2日两次咽拭子或痰或便SARS-Cov2核酸检测阴性。

### 3.12.1筛选访视

当获得受试者的书面知情同意后，研究者可对受试者进行筛查。各研究中心的研究者通过登陆EDC系统，录入所有进入筛查流程的潜在受试者信息。筛查访视应在2天时间窗内完成。

筛查访视时将收集以下资料：

（1）人口学信息：内容包括性别、年龄等。

（2）病史询问：筛查时应详细询问受试者伴随基础病史，合并严重的心、肝、肾脏疾病或血液、内分泌、风湿免疫、神经、恶性肿瘤等系统性疾病，且未稳定控制者将不被纳入。

（3）前次COVID-19诊疗情况及流行病学调查，包括起病日期、出院诊断、首次病毒核酸阳性时间、住院期间治疗情况（抗病毒、激素、恢复期血浆、出院后服用药物及接触史。

（4）症状：记录病人入院时的症状，主要包括肌痛、头痛、腹痛、关节痛、腹泻、虚弱、神经性症状（包括厌食、恶心、呕吐、眩晕、意识模糊、震颤、惊厥、昏迷等）、呼吸道症状（包括咳嗽、胸闷、气促、胸痛、呼吸困难、急性肺损伤/急性呼吸窘迫综合征等）等。

（5）生命体征及体格检查：内容包括体温、血压、心率、指端脉氧检查、呼吸频率等。

（6）COVID-19病毒核酸定量检测（探针法绝对定量qRT-PCR）：痰、咽拭子、血液、粪便等标本COVID-19核酸检测阳性患者才考虑入组。

（7）血液检查：内容包括血常规、肝肾功能（谷丙转氨酶、谷草转氨酶、肌酐、白蛋白、乳酸脱氢酶）、电解质（钾、钠、钙离子）、淋巴细胞亚群分析、免疫球蛋白，心肌酶，凝血功能、血气分析、C反应蛋白、降钙素原、细胞因子（TNF-α、IL-2、IL-4、IL-6、IFN-α、IFN-β、IFN-γ等）。

（8）尿液检查：内容包括尿常规和尿妊娠检查。

（9）大便常规。

（10）影像学检查：进行肺部CT和X线检查。

（11）合并用药：询问受试者合并用药情况，并记录于电子病例报告表中。

### 3.12.2治疗期访视

治疗期访视时将收集以下资料：

（1）症状（治疗1、3、5、7、10、14天）：记录受试者症状，主要包括肌痛、头痛、腹痛、关节痛、腹泻、虚弱、神经性症状（包括厌食、恶心、呕吐、眩晕、意识模糊、震颤、惊厥、昏迷等）、呼吸道症状（包括咳嗽、胸闷、气促、胸痛、呼吸困难、急性肺损伤/急性呼吸窘迫综合征等）等。

（2）生命体征及体格检查（治疗1、3、5、7、10、14天）：内容包括体温、血压、心率、指端脉氧检查、呼吸频率等。

（3）COVID-19病毒核酸定量检测（探针法绝对定量qRT-PCR）（治疗1、3、5、7、10、14天）：包括痰、咽拭子、血液、粪便等标本的核酸检测。

（4）血液检查：内容包括治疗1、7、10、14天时血常规、肝肾功能（谷丙转氨酶、谷草转氨酶、肌酐、白蛋白、乳酸脱氢酶）、电解质（钾、钠、钙离子）、心肌酶，凝血功能、血气分析、C反应蛋白、降钙素原；治疗1、3、5、7、10、14天时细胞因子、淋巴细胞亚群分析；治疗1、7、14天时免疫球蛋白。

（5）尿液检查：内容包括治疗1、7、10、14天时尿常规。

（6）治疗1、7、10、14天时大便常规。

（7）影像学检查：治疗1、7、14天时进行肺部CT和X线检查。

（8）合并用药：治疗1、3、5、7、10、14天时询问受试者合并用药情况，并记录于电子病例报告表中。

（9）不良事件：自患者签署知情同意书后，每次访视均应询问和记录不良事件，并按照CTCAE 5.0版进行分级。

### 3.12.3随访期访视

治疗结束后第14天、28天随访期访视时将收集以下资料：

（1）症状：记录受试者症状，主要包括肌痛、头痛、腹痛、关节痛、腹泻、虚弱、神经性症状（包括厌食、恶心、呕吐、眩晕、意识模糊、震颤、惊厥、昏迷等）、呼吸道症状（包括咳嗽、胸闷、气促、胸痛、呼吸困难、急性肺损伤/急性呼吸窘迫综合征等）等。

（2）生命体征及体格检查：内容包括体温、血压、心率、指端脉氧检查、呼吸频率等。

（3）COVID-19病毒核酸定量检测（探针法绝对定量qRT-PCR）：包括痰、咽拭子、血液、粪便等标本的核酸检测。

（4）血液检查：血常规、肝肾功能（谷丙转氨酶、谷草转氨酶、肌酐、白蛋白、乳酸脱氢酶）、电解质（钾、钠、钙离子）、心肌酶，凝血功能、血气分析、C反应蛋白、降钙素原、细胞因子、淋巴细胞亚群分析、免疫球蛋白。

（5）尿常规检查。

（6）大便常规。

（7）影像学检查：肺部CT和X线检查。

（8）合并用药：询问受试者合并用药情况，并记录于电子病例报告表中。

（9）不良事件：每次访视均应询问和记录不良事件，并按照CTCAE 5.0版进行分级。

# 四、数据管理和质量保证

本试验采用电子病例报告表（eCRF）模式，在试验启动之前对试验参加人员进行相关培训。

（1）eCRF构建及审查：数据管理员根据 “研究病历”构建eCRF。构建完成后交研究者和申办者审查。一致通过后数据管理员根据研究者提供的信息创建帐号。

（2）EDC系统测试：对于构建好的eCRF进行试验前测试，确保无误并记录。

数据的录入：临床研究者应指定数据录入员，在受试者访视后，录入员应及时、准确地将研究病历中的数据录入到eCRF上，并输入电子签名（即帐号密码）。

监查员确认所有电子病例报告表填写的完整，并与原始资料一致，发现有错误的地方及时更正并电子签名。

（3）数据疑问及答疑：对eCRF中存在的疑问，监查员将随时在线提出疑问，研究者应尽快在线给予解答，修改错误数据，必要时监查员可以重复发出疑问。疑问及解答的交换应当采用疑问表形式，疑问表应保存备查。

（4）数据锁定及导出：在每一位受试者完成试验并经监查员审核无误后，由数据管理员进行数据逻辑核查和医学核查，若发现病例报告、严重不良事件，以及实验室数据存在疑问，数据管理员将产生对数据向研究者发出询问，在所有疑问均得到解决并确认建立的数据库正确后进行数据锁定，并完成数据管理审核报告。数据全部锁定后，由数据管理员导出数据库，交统计人员进行统计分析。

# 五、统计分析

## 5.1样本量的确定

预估总共纳入210例受试者，其中试验组140例，对照组70例。

## 5.2统计分析集

统计分析将用到3个分析集：全分析集（FAS）、符合方案集（PPS）和安全性分析集（SS）。同时采用FAS和PPS对所有疗效指标进行分析，如二者结果不一致，将进一步分析其原因。使用FAS进行分析时，对缺失数据将采用LOCF法（末次数据填补）进行数据填补。SS将用于安全性分析。

（1）全分析集（FAS）：包括所有符合入排标准进入研究，至少使用1次研究药物并有至少1次用药后疗效评价的对象。违背入排标准的病例将不包括在内。

（2）符合方案集（PPS）：包括所有符合入排标准进入研究，按照方案要求完成全部随访，没有重大方案违背，依从性良好（用药依从性≥80%）的对象。

（3）安全集（SS）：指所有进入研究，至少使用过1次研究药物并且有用药后安全性评价的病例。

## 5.3统计分析方法

**（1）一般原则**

除特别指出外，所有统计检验均为双侧检验，P<0.05即可认为所判断的差异有统计学意义。

- 定量数据：采用例数、算术均数、标准差、中位数和范围描述。
- 定性数据：采用频数、构成比或百分比描述。
- 统计检验：首先考虑用参数统计方法，如果数据分布与检验假设的要求相差较大，则用非参数统计方法。

数据库与统计分析: 数据库为epidata形式，用SAS 9.2统计软件进行统计分析。

**（2）病例特征**

- - 入组及完成情况：总结各中心入组及完成病例数，列出脱落病例的清单。
  - 一般信息的基线特征：基线定义为病例筛选期所获得的数据。对患者的人口学特征、病史等进行描述。

**（3）疗效评价**

- 疗效相关参数的基线评价: 与疗效评价相关的基线数据，定义为病例筛选期所获得的数据。采用FAS进行基线评价。分类变量采用CMH-χ2检验、Fisher’s精确概率法或等级数据的Wilcoxon秩和检验比较组间的差别。连续变量采用成组 t 检验或Wilcoxon秩和检验。分类疗效指标，如影像学评估资料的组间比较采用CMH-χ2检验或Logistic回归。

**（4）安全性评价**

- 对各治疗组的不良事件、严重不良事件分别统计发生例数，类别和严重程度；
- 采用CMH-χ2检验或Fisher’s精确概率法比较二组不良事件、不良反应的发生率；
- 采用成组t检验或Mann-Whitney法比较二组治疗后为连续变量的检查指标；
- 统计描述二组治疗后实验室指标正常、异常变化或异常加重的比例。

**（5）缺失数据处理**

对于受试者失访导致数据缺失，计划采用死亡或失访前所有可获得信息。主要结局分析时假设数据为随机缺失，并在敏感性分析中探讨失访是否与分析的结局相关。通过使用既往访视中得到的数据对失访进行预测来调查该问题的严重性，确定能够预测失访的变量将被纳入至预测模型中。此外，采用多重填补技术对主要和次要结局进行敏感性分析。

# 六、受试者保护：知情同意、收益风险、保密、利益冲突

## 6.1伦理方面的考虑

临床试验必须遵循赫尔辛基宣言和我国有关临床试验研究规范、法规进行。在试验开始之前，由临床试验负责单位的伦理委员会批准该试验方案后方可实施临床试验。

每一位受试者入选本研究前，研究医师有责任以书面文字形式，向其或其指定代表人完整、全面地介绍本研究的目的、程序和可能的风险。应让受试者知道他们有权随时退出本研究。入选前必须给每位受试者一份书面知情同意书，研究医师有责任让每位受试者在进入研究之前获得知情同意，知情同意书应作为临床试验文档保留备查。

## 6.2试验方案的审批

临床研究开始前向伦理委员会提供临床试验方案、详细的病人信息页和知情同意书的副本文件，以获得实施该临床研究的独立批准文件。如果后续方案有修正，将送交伦理委员会再次审核。

## 6.3知情同意过程和知情同意书文本

由研究者向受试者详细介绍本试验的背景、目的、步骤、获益、风险等情况，并解答受试者提出的试验相关的问题。

受试者充分了解本试验的背景、目的、步骤、风险及获益情况，对提出的问题得到答复后，在自愿参加本研究的情况下签署知情同意书。

知情同意书（另附）。

## 6.4受试者获益和风险

本研究将为所有受试者购买保险。

参与本次研究不能保证会从中获得临床益处。但是，从本次研究中获得的信息有利于将来应用法匹拉韦对复阳患者进行治疗。本研究将为试验组受试者免费提供法匹拉韦片，治疗时间最长14天。

鉴于研究药物法匹拉韦片在既往研究中观察到的安全性良好，预期其治疗COVID-19病毒核酸复阳的患者，不良反应风险可控。

## 6.5保密声明

本方案中所包含的所有信息的所有权归申办方所有，仅提供给研究者、合作研究者、伦理委员会和监督管理部门等相关机构和人员审阅。在未得到申办方书面批准情况下，除了在与可能参加本试验的受试者签署知情同意书时，向其做必要的解释外，严禁将任何信息告知与本试验无关的第三方。

# 七、不良事件、严重不良事件的评估及处理

## 7.1不良事件观察

不良事件（AE）定义：AE指临床研究对象在接受治疗方案后出现的任何不利的医学事件，但不一定与治疗有因果关系。

AE可以是和使用医学产品有时间上的联系的任何不愉快的或者和使用产品目的无关的体征（包括异常的实验室检查结果）、症状或疾病，不论是否认为与医学产品有关。

## 7.2 AE分级

AE按NCI《常见急性及亚急性毒性分级标准》（NCI-CTCAE 5.0）分为0-5级：

1级：轻度；无症状或轻微；仅为临床或诊断所见；无需治疗。

2级：中度；需要较小、局部或非侵入性治疗；与年龄相当的工具性日常生活活动（做饭、购买衣物、使用电话、理财等）受限。

3级：严重或者具重要医学意义但不会立即危及生命；导致住院或者延长住院时间；致残；自理性日常生活活动（洗澡、穿脱衣、吃饭、盥洗、服药等，并未卧床不起）受限。

4级：危及生命；需要紧急治疗。

5级：与AE相关的死亡。

如NCI毒性分级标准未列出的AE，可根据以下标准判断：

Ⅰ度（轻微）：有不舒服的感觉，但不影响正常的日常活动；

Ⅱ度（中度）：不舒服的程度达到足以减少或影响正常的日常活动；

Ⅲ度（严重）：不能工作或正常的日常活动；

Ⅳ度（致命）：致残或致死。

## 7.3 AE与研究药物之间关系的判断

按“肯定有关、很可能有关、可能有关、可能无关、无关”五级分类法对AE和研究药物之间可能存在的关联作出评估（见表1）。前三级判断为与药物相关。不良事件发生率计算时将三者合计作为分子，用于评价安全性的全部受试者例数作为分母。

表1：AE与治疗方案的关系判定标准

| **标准** | **肯定有关** | **很可能有关** | **可能有关** | **可能无关** | **无关** |
| --- | --- | --- | --- | --- | --- |
| 合理的时间顺序 | 是 | 是 | 是 | 是 | 否 |
| 已知的药物反应类型 | 是 | 是 | 是 | 否 | 否 |
| 去除原因可以改善 | 是 | 是 | 是或否 | 是或否 | 否 |
| 再次给药可重复出现 | 是 | ？ | ？ | ？ | 否 |
| 反应可能有另外解释 | 否 | 否 | 否 | 是 | 是 |

## 7.4 AE的记录

详细记录研究期间出现的各种AE名称、严重程度、出现时间、持续时间、处理措施、转归以及与治疗的关系等，并如实填写CRF。异常的实验室检查数据记录在CRF中，并重复该项检查每周至少一次，随访至恢复正常或研究结束。

## 7.5 严重不良事件的报告和处理

（1）严重不良事件的定义

严重不良事件（SAE）是指临床研究过程中发生的需要住院治疗或延长住院时间、伤残、影响工作能力、危及生命或死亡、导致先天畸形等医学事件。包括以下非预期医学事件：

- 导致死亡的事件；
- 危及生命的事件（定义为受试者在事件发生时有死亡危险）；
- 需要住院治疗或延长住院时间的事件；
- 可导致永久性或严重残疾/功能不全的事件；
- 先天异常或出生缺陷。

（2）住院治疗

临床研究中导致住院治疗或住院时间延长的不良事件应视为严重不良事件。任何初次被医疗机构收住院（即使短于24小时）的情况均符合此标准。

住院不包括以下情况：

- 康复机构
- 疗养院
- 常规急诊室收治
- 当日手术（如门诊/当日/非卧床的手术）

与不良事件恶化无关的住院治疗或住院时间延长本身不是严重不良事件，例如：

- 因原有疾病入院，并没有新的不良事件的发生，也没有原有疾病的加重（如：为了检查研究前至今持续存在的实验室检查异常）；
- 管理原因的住院；
- 临床研究期间研究方案规定的住院；
- 与不良事件恶化无关的择期住院；
- 已预定的治疗或外科手术应在整个研究方案和/或受试者个人的基线资料中予以记录；
- 仅因为血液制品使用而入院。

诊断性或治疗性的侵入性（如手术）、非侵入性操作不应作为不良事件报告。但导致此项操作的疾病状况符合不良事件的定义时，应予以报告，如不良事件报告期间发生的急性阑尾炎应报告为不良事件，而因此进行的阑尾切除术应记录为该不良事件的治疗方法。

（3）SAE的报告程序

严重不良事件的报告应自受试者签署知情同意书开始，直至最后一次使用研究药物后的30个公历日（含第30天）。末次给药30天后发生的严重不良事件，除非怀疑与研究药品有关，一般不予报告。

试验期间，若发生严重不良事件必须在24小时内报告给临床监查员和主要研究者，同时填写《严重不良事件（SAE）报告表》，签名及注明日期，并以传真的形式立即上报申办单位、组长单位、研究单位伦理委员会、国家食品药品监督管理总局（CFDA）及研究者所在地区的（省或市）的食品药品监督管理局。

严重不良事件应详细记录症状、严重程度、发生时间、处理时间、采取措施、随访时间和方式以及转归情况。如果研究者认为某严重不良事件与试验药物无关，而与研究条件（例如终止原治疗，或试验过程中的合并症）潜在相关，则这种关系应在病历报告表的严重不良事件页的叙述部分详细说明。如果某种正在发生的严重不良事件的强度或其与受试药物的关系发生改变，应立即将严重不良事件随访报告送交申办者。所有的严重不良事件均应随访至恢复或稳定。

# 八、研究质量控制

质量控制是本项研究的关键。质量控制体现在受试者筛查和招募、基线数据获取、随访数据获取、结局定义和评估等各个研究环节。通过设置质量控制委员会，制定质量控制方案，进行质量控制评估并将结果反馈至各研究中心或研究其他部门，以维持和提高整个试验过程研究数据的质量。

## 8.1 研究培训

人员培训和操作流程的预先检验对于操作流程的标准化以及保证数据质量至关重要。基于研究方案对研究人员进行详细培训。研究方案是进行标准化研究流程的一个重要规范和参考。要求所有研究人员参加研究集中性或区域性培训课程。除外研究启动前集中培训及研究过程中定期开展研究培训外，研究质量控制或质量稽查人员可定期或按需前往研究中心对研究人员进行现场培训。

## 8.2 数据核查

研究负责单位将负责数据核查，包括对丢失数据、不真实值的核对，对不一致数据的交叉核对，以及对数据的抽查核对。所有数据问题将及时返还给数据录入/转换员以便及时处理。数据核查人员在网上生成数据质询报告，这些报告总结了研究数据质询的数目和类型。数据录入/转换员和研究中心的研究人员负责及时复审以及解决数据问题。

## 8.3 质量控制报告

质量控制委员会使用质量评估指标记录数据质量并给各个研究中心提供反馈，进一步在质量控制报告中持续跟踪这些质量评估指标。质量控制委员会将生成质量控制报告分发至各研究中心，并进行定期电话联系或研究中心随访讨论，确保各研究中心研究质量。

# 九、研究进度安排

20200326-20200401:完成方案设计、伦理审批、药品准备、CRO公司等相关工作；

20200401-20200601:完成病例筛选入组；

20200601-20200615:完成治疗期访视；

20200615-20200715:完成受试者随访；

20200715-20200915:关闭研究、整理数据、撰写研究报告。

**方案签字页**

我作为参与研究的医生/统计分析人员，已经阅读过这项研究的方案。

我已经与研究负责人充分讨论了这项研究的目的和本方案的内容。

我同意根据本方案进行研究，并遵守其要求，遵守伦理规范，并在药物临床研究质量管理规范（GCP）指导下开展本项临床研究。

我同意对本研究方案的内容保密，不会透露给第三方，且方案的内容仅用于进行这项研究。

我理解，如果本项研究在任何时间以无论什么原因作出提前终止或暂停这项研究的决定，都将以书面形式通知我。同样，如果我决定退出执行这项研究，也会以书面形式立即通知本研究负责单位及主要研究者。

| 研究中心： |  |
| --- | --- |
| 研究者签名： |  |
| 地址： |  |
| 电话： |  |
| 日期： | 年 月 日 |
